# Comparison of minimally invasive surgery and open surgery following chemotherapy for cStage IVb gastric cancer: a multicenter retrospective cohort study

**DOI:** 10.1007/s10120-026-01779-y

**Published:** 2026-07-20

**Authors:** Yusuke Fujii, Shigeo Hisamori, Nobuaki Hoshino, Seiichiro Kanaya, Eiji Tanaka, Yoshito Yamashita, Akira Miki, Kosuke Toda, Michihiro Yamamoto, Yosuke Kinjo, Dai Manaka, Hiroaki Hata, Hironori Kawada, Shotaro Matsuda, Atsushi Itami, Kyoichi Hashimoto, Kenjiro Hirai, Sanae Nakajima, Hiroshi Okabe, Masazumi Sakaguchi, Yuichiro Kawamura, Takashi Sakamoto, Shintaro Okumura, Tatsuto Nishigori, Shigeru Tsunoda, Kazutaka Obama

**Affiliations:** 1 Department of Surgery, Kyoto University Hospital, 54 Shogoin Kawahara-cho, Sakyo-ku, Kyoto, 606-8507 Japan; 2 Kyoto Esophageal and Gastric Surgery Group, Kyoto, Japan; 3 Department of Gastroenterological Surgery, Japanese Red Cross Osaka Hospital, Osaka, Japan; 4 Department of Gastroenterological Surgery, Kitano Hospital, Osaka, Japan; 5 Department of Gastroenterological Surgery, Japanese Red Cross Wakayama Medical Center, Wakayama, Japan; 6 Department of Gastroenterological Surgery, Toyooka Hospital, Toyooka, Japan; 7 Department of Surgery, Shiga General Hospital, Moriyama, Japan; 8 Department of Gastroenterological Surgery, Tenri Hospital, Tenri, Japan; 9 Department of Gastroenterological Surgery and Oncology, Himeji Medical Center, Himeji, Japan; 10 Department of Surgery, Gastrointestinal Center, Kyoto Katsura Hospital, Kyoto, Japan; 11 Department of Surgery, National Hospital Organization, Kyoto Medical Center, Kyoto, Japan; 12 Department of Gastroenterological Surgery, Hyogo Prefectural Amagasaki General Medical Center, Amagasaki, Japan; 13 Department of Surgery and Transplant Surgery, Kobe City Medical Center General Hospital, Kobe, Japan; 14 Department of Surgery, Kobe City Nishi-Kobe Medical Center, Kobe, Japan; 15 Department of Gastroenterological Surgery, Uji-Tokushukai Medical Center, Uji, Japan; 16 Department of Surgery, Japanese Red Cross Otsu Hospital, Otsu, Japan; 17 Department of Gastroenterological Surgery, Kobe City Medical Center West Hospital, Kobe, Japan; 18 Department of Gastroenterological Surgery, New Tokyo Hospital, Matsudo, Japan; 19 Department of general Surgery, Kyoto City Hospital, Kyoto, Japan; 20 Department of Surgery, Kokura Memorial Hospital, Kitakyushu, Japan

**Keywords:** Minimally invasive surgical procedures, Robotic surgical procedures, Laparoscopy, Stomach neoplasms, Neoplasm metastasis

## Abstract

**Background:**

The standard treatment for stage IV gastric cancer (GC) is chemotherapy, but advances in systemic therapy have rendered curative surgery a viable option. However, the role of minimally invasive surgery (MIS) in this context remains unclear. This multicenter retrospective cohort study aimed to evaluate safety and effectiveness of MIS post-chemotherapy for patients with cStage IVb GC.

**Methods:**

Patients with cStage IVb GC who underwent curative-intent MIS or open surgery post-chemotherapy at 19 institutions between 2011 and 2022 were reviewed. Propensity score-matching was performed to adjust for confounding variables. The primary outcome was postoperative complications. Secondary outcomes were perioperative outcomes and long-term survival.

**Results:**

Among 237 eligible patients (MIS, 130; open surgery, 107), 64 matched pairs were analysed. The incidence of Clavien–Dindo grade ≥ II complications was 31.2% in the MIS group and 46.9% in the open group, with no significant difference (risk ratio 0.67, 95% confidence interval [CI] 0.41–1.08). MIS was associated with a longer operative time (mean difference [MD] 98 min, 95% CI 53–143) but reduced blood loss (MD, − 550 g; 95% CI, − 738 to − 362). The MIS group achieved R0 resections more often (92% vs. 78%). The 3-year overall survival rates were 56.5% and 44.6%, respectively (hazard ratio 0.83, 95% CI 0.51–1.36).

**Conclusions:**

In curative-intent gastrectomy following chemotherapy for cStage IVb gastric cancer, MIS was not associated with worse perioperative outcomes or long-term survival compared with open surgery. Given its less invasive nature, MIS could be considered a feasible option for these patients when performed by experienced surgeons.

**Supplementary information:**

The online version contains supplementary material available at https://doi.org/10.1007/s10120-026-01779-y.

## Introduction

Despite advances in systemic therapy, stage IV gastric cancer (GC) has a poor prognosis. Contemporary systemic chemotherapy has extended the median overall survival (OS) to 17–18 months, but durable disease control is achieved in only a few patients [1, 2]. The advent of conversion surgery, defined as curative-intent resection following marked tumor regression induced by chemotherapy, has ushered in a potential paradigm shift for select patients with stage IV GC [3]. Several retrospective series have reported 3-year survival rates > 50% among patients who attained R0 resection after systemic chemotherapy [4–8]. Although the phase III RENAISSANCE trial failed to demonstrate survival benefit of conversion surgery, the ongoing JCOG2301 trial is expected to further evaluate its survival impact [9, 10].

However, conversion surgery presents technical challenges. Chemotherapy-induced fibrosis can obliterate normal tissue planes, thereby increasing operative difficulty and the risk of morbidity [11]. Postoperative complications or decreased performance status may further delay or hamper continuation of systemic therapy, the very treatment that renders the disease resectable [12]. Minimally invasive surgery (MIS), with its superior magnification and reduced physiologic stress, offers an attractive strategy to overcome these obstacles.

Previously, we reported on the feasibility of MIS for advanced GC and several randomised controlled trials have provided validating evidence [13–18]. However, the evidence for MIS in conversion surgery settings is fragmented. Published cohorts are small, single-institutional, the experience level of operating surgeons is seldom standardised, and differences in the surgical era are often not accounted for [12, 19]. Consequently, the comparative safety and oncologic adequacy of MIS versus open gastrectomy in stage IV GC remain unclear [20].

Therefore, *we conducted a multicenter retrospective study across institutions with maintained surgical quality*, *using robust statistical methodology*,* to compare perioperative and survival outcomes between MIS (laparoscopic or robotic) and open surgery in patients initially diagnosed with stage IV GC* *who underwent curative-intent gastrectomy after systemic chemotherapy.*

## Methods

### Study design and patient cohort

This multicenter retrospective cohort study, approved by the Ethics Committee of Kyoto University (approval number: R4116), was conducted in accordance with Strengthening the Reporting of Observational Studies in Epidemiology (STROBE) reporting guidelines [21].

We included adults aged ≥ 18 years who underwent curative-intent surgery after chemotherapy for initially diagnosed cStage IVb histologically proven gastric or gastroesophageal junction (Siewert Type 2 or 3) adenocarcinoma, between January 2011 and December 2022. Data were collected from Kyoto University Hospital and 18 institutions affiliated with the Kyoto Esophageal and Gastric Surgery Study Group (KEGG), who regularly conduct study sessions on surgical techniques to ensure overall surgical quality across institutions. Patients with remnant GC, recurrent cases, those who underwent simultaneous resection for other primary cancers, or those who underwent surgery for palliative care were excluded. Cancer staging was performed according to the 15th edition of the Japanese Classification of Gastric Carcinoma [22, 42].

### Indications for surgical resection, follow-up

Surgical indications were determined at each institution, where surgical quality was ensured, in accordance with the gastric cancer treatment guidelines, and based on multidisciplinary consensus among surgeons and medical oncologists that R0 resection was achievable and oncologically reasonable after chemotherapy [42]. The duration of chemotherapy and the timing of surgery were determined at the discretion of each institution. Combined resection of adjacent organs was performed when deemed necessary to achieve R0 resection based on preoperative computed tomography findings, or when intraoperative assessment indicated that achieving a clear margin through dissection from adjacent organs would be difficult.

Postoperative follow-up was left to the discretion of each institution; however, in principle, it was conducted as follows based on guideline recommendations for advanced gastric cancer: patients who achieved R0 resection received 6–12 months of adjuvant chemotherapy, followed by outpatient follow-up every 3 months, whereas those with non-R0 resection resumed systemic chemotherapy with closer follow-up.

### Exposure

The exposure of interest was the surgical approach (MIS or open). MIS was defined as a surgery performed using either laparoscopy or robotic assistance. Patients who underwent MIS but who then required conversion to open surgery were included in the MIS group.

### Outcomes

The primary outcome was postoperative complications (Clavien–Dindo [CD] classification grade ≥ II) [24]. Secondary outcomes included perioperative outcomes in relation to 30-day mortality, operative time, blood loss volume, number of dissected lymph nodes, time to oral intake, postoperative hospital stays, readmission within 30 days after discharge, reoperation owing to complications, proportion of R0 resection, proportion of postoperative chemotherapy administration, days from operation to postoperative chemotherapy; as well as long-term outcomes in relation to progression-free survival (PFS) and OS. For the survival data, the date of curative surgery was set as the index date, OS was defined as the time to death from any cause, and PFS was defined as the time to death from any cause or the first progression/recurrence.

### Covariates

The covariates used to calculate the propensity score (PS) included important patient, tumor, and operative variables. These variables were determined through discussions among the multiple authors based on their clinical expertise and literature reviews.

The patient-related variables included age, sex, body mass index (< first quartile [19 kg/m^2^], > first quartile and < third quartile [23.3 kg/m^2^], or ≥ third quartile), comorbidity (Charlson Comorbidity Index = 0, 1, or ≥ 2), American Society of Anesthesiologists Physical Status (≤ 2 or ≥ 3), and prognostic nutritional index (PNI) (≥ 40 or < 40) [25]. PNI was calculated using the formula: PNI = 10 × serum albumin (g/dL) + 0.005 × lymphocytes (/mm^3^), which reflects nutritional and immunological status [26, 27].

Tumor-related variables included clinical T-category (≤ T3 or ≥4a), clinical N-category (positive or negative), clinical P-category (positive or negative), clinical CY-category (positive or negative), clinical H-category (positive or negative), macroscopic type (type 4 or others), Lauren type (intestinal or diffuse), and clinical response evaluation of preoperative chemotherapy according to RECIST guideline version 1.1 (progressive disease [PD], stable disease + non-CR/non-PD, or complete response [CR] + partial response [PR]) [28].

The operative variables included the procedure (total gastrectomy [TG] or non-TG), implementation of para-aortic lymph node dissection (PAND) (yes or no), combined resection of major organs (yes or no), and operative year (2011–2016 or 2017–2022). The major organs included the esophagus, spleen, pancreas, liver, and colon.

### PS-matched cohort

We applied PS matching to mitigate selection bias and confounding [29]. Each patient’s propensity to undergo MIS was calculated using multivariable logistic regression accounting for all potential confounding and prognostic factors, which are listed in the *Covariates* Sect [30]. The patients were matched 1:1 using a nearest-neighbour algorithm without replacement. A calliper was set to 0.2 standard deviations of the logit of the PS [31, 32]. Covariate balance was evaluated using the absolute standardised mean difference (ASMD) [33]. An ASMD < 0.1 was considered a negligible difference between the groups [34].

### Statistical analysis

We first visualised the chronological trends in the operative approaches (open, laparoscopic, and robotic). We then described baseline characteristics and preoperative chemotherapy. Categorical variables are reported as absolute numbers (n) and proportions (%). Continuous variables are reported as medians and interquartile ranges. Subsequently, we compared perioperative outcomes, pathological findings, survival outcomes, and types of first recurrence or progression in the matched cohort. Comparisons of perioperative and survival outcomes in the matched cohort were performed using generalized estimating equations with robust standard errors, taking pairwise correlation into account [35]. For categorical outcome variables, risk ratios (RRs) were calculated using binomial or modified Poisson regression with robust variance. For continuous outcome variables, mean differences (MDs) were calculated using linear regression. Survival probability was described using the Kaplan–Meier method, and hazard ratios (HRs) were calculated using Cox regression. Missing data were infrequent (< 5% for any variable). We assumed the missing data mechanism to be missing completely at random and performed a complete case analysis.

In addition, we conducted subgroup analyses to assess whether the effect size for postoperative complications and OS differed among subgroups based on age (< 75 and ≥ 75), sex, macroscopic type, Lauren type, clinical response by RECIST, cT, cP, cCY, cH, procedure, PAND, combined resection, operative year, and neoadjuvant/ conversion setting. For stage IV cancer, the term “neoadjuvant chemotherapy (NAC)” is considered appropriate for patients whose tumors are technically resectable at initial diagnosis, distinguishing them from conversion surgery. Yoshida et al. classified stage IV cancer into four categories; accordingly, we defined Yoshida category 1 as a NAC setting and categories 2–4 as a conversion setting in this study [3]. Furthemore, considering the difference in the distribution of pathological findings and R0 status between the two groups, we additionally performed subgroup analyses based on pathological metastasis and R0 status.

To assess the robustness of our findings, we conducted several sensitivity analyses as follows: First, we performed an analysis excluding patients who underwent pancreaticoduodenectomy. This was because these patients were considered to be at higher risk for postoperative complications and may have experienced different types of complications. In addition, as previous reports have suggested that the duration of preoperative chemotherapy may influence prognosis, we also conducted an analysis including this variable in the propensity score model [8, 36]. We additionally conducted an analysis including three variables reflecting the detailed extent of distant metastases at initial diagnosis (distant lymph node metastasis, the number of liver metastases, and the extent of peritoneal dissemination) in the propensity score model. Moreover, we conducted an assessment using inverse probability of treatment weighting (IPTW) with the Average Treatment effect on the Treated (ATT) estimand. In the main analysis, we did not consider institutional factors, but there may be correlations within institutions regarding patient backgrounds, selection of surgical approach, and indications for curative surgery. Therefore, we conducted an additional PS matching analysis, incorporating hospital volume factors (high ≥ third quartile; low < third quartile) determined by the annual number of gastrectomies. We also applied the generalized estimating equations (GEE) to account for intra-institutional correlations [37]. Furthermore, we conducted another GEE analysis with multiple imputations by chained equations, assuming the missing data mechanism to be missing at random (20 imputations). Data analyses were performed using the “MatchIt”, “WeightIt”, and “MICE” packages in R version 4.2.2 (R Foundation for Statistical Computing, Vienna, Austria) and “xtgee” package in STATA version 17.0 (StataCorp, College Station, Texas, USA) software.

## Results

### Trends in surgical approaches

Over the study period, the number of patients who underwent MIS steadily increased, surpassing open surgeries between 2015 and 2016. In recent years, open surgeries have been consistently performed and robotic surgeries have become comparable in number to laparoscopic surgeries (Figure S1).

### Study cohort

**Fig. 1:**
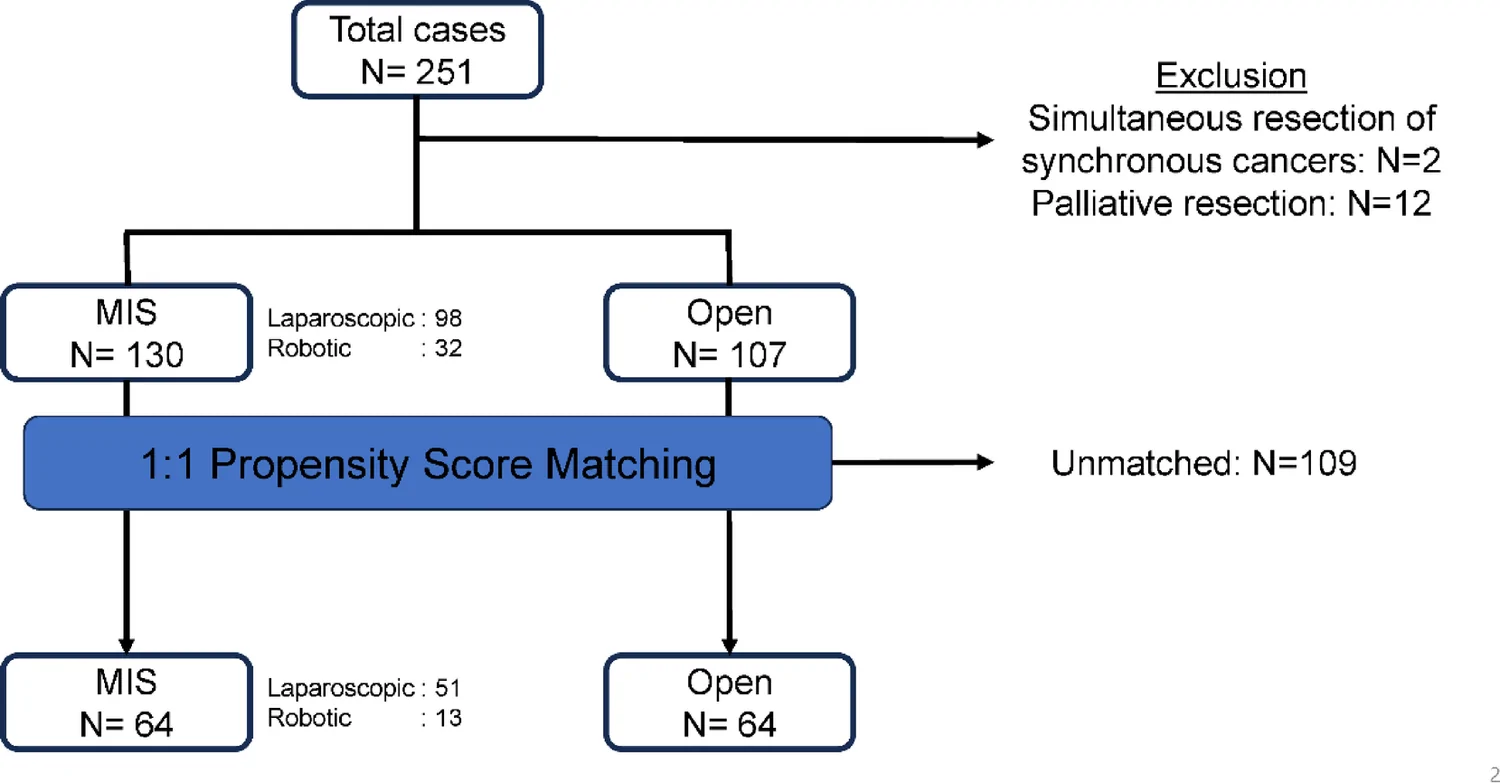
Flow diagram of patient selection and propensity score matching MIS, minimally invasive surgery

In total, 251 patients met the inclusion criteria. After excluding two patients who underwent simultaneous resection of synchronous cancers and 12 who underwent palliative resection, 237 patients (MIS: 130; open surgery: 107) were included in the analysis. Using 1:1 PS matching, 64 patients were assigned to each group (Fig. 1).

Patient characteristics before and after matching are shown in Table 1. In the entire cohort, the open surgery group had a higher proportion of macroscopic type 4 cases, earlier operative periods, more frequent PAND, and combined resections. In the matched cohort, 21 patients in the MIS group and 20 in the open group had distant lymph node metastasis at initial diagnosis; ultimately, distant (para-aortic) lymph node dissection was performed in 12 (57%) and 11 (55%) patients, respectively. Among patients with liver metastases, liver metastasectomy was ultimately performed in 2 of 10 patients (20%) in the MIS group and 7 of 11 patients (64%) in the open group. Among patients with peritoneal dissemination, resection of peritoneal metastatic lesions was ultimately performed in 4 of 21 patients (19%) in the MIS group and 3 of 21 patients (14%) in the open group. After matching, all covariates, except for age, had an ASMD < 0.1, indicating a well-balanced covariate distribution. While the ASMD for age was 0.106, which slightly exceeded 0.1, it was considered acceptable.

**Table 1:** Baseline characteristics in patients undergoing curative-intent gastrectomy by MIS or Open

		Entire cohort (*N* = 237)					Matched cohort (*N* = 128)				
		MIS (*N* = 130)		Open (*N* = 107)			MIS (*N* = 64)		Open (*N* = 64)		
						ASMD					ASMD
Age (years)		69	(63–74)	68	(62–72)	0.140	68	(62–75)	68	(62–72)	0.106
Sex	Male	87	(67%)	77	(72%)	0.087	42	(66%)	44	(69%)	0.066
BMI (kg/m^2^)											
	≤ 19	34	(26%)	29	(27%)	0.023	20	(31%)	18	(28%)	0.071
	> 19, < 23.3	63	(48%)	53	(50%)	0.001	28	(44%)	29	(45%)	0.031
	≥ 23.3	33	(25%)	25	(23%)	0.028	16	(25%)	17	(27%)	0.036
Charlson comorbidity index*											
	0	84	(65%)	65	(61%)	0.054	40	(63%)	40	(63%)	0.000
	1	31	(24%)	31	(29%)	0.060	17	(27%)	16	(25%)	0.036
	≥2	15	(12%)	11	(10%)	0.001	7	(11%)	8	(13%)	0.050
ASA-PS	≥ 3	13	(10%)	10	(9%)	0.008	7	(11%)	8	(13%)	0.052
Prognostic nutritional index (PNI)	≥ 40	45	(41–48)	45	(41–49)	0.088	46	(42–48)	46	(41–49)	0.059
	Missing	1	(1%)	5	(5%)						
Macroscopic type	Type 4	22	(17%)	27	(25%)	0.255	16	(25%)	14	(22%)	0.084
Lauren type						0.103					0.032
	Intestinal	56	(43%)	42	(39%)		26	(41%)	27	(42%)	
	Diffuse	72	(55%)	64	(60%)		38	(59%)	37	(58%)	
	Missing	2	(2%)	1	(1%)						
cT						0.288					0.000
	≤ 3	29	(22%)	36	(34%)		19	(30%)	19	(30%)	
	≥ 4a	101	(78%)	71	(66%)		45	(70%)	45	(70%)	
cN	positive	118	(91%)	96	(90%)	0.083	56	(88%)	55	(86%)	0.056
Distant lymph node metastasis† (at initial diagnosis)	positive	43	(33%)	48	(45%)		21	(33%)	20	(31%)	
	limited to a2/b1	30	(23%)	31	(29%)		15	(23%)	14	(22%)	
	within a1/b2	10	(8%)	9	(8%)		4	(6%)	3	(5%)	
	beyond a1/b2	3	(2%)	8	(7%)		2	(3%)	3	(5%)	
cH	positive	19	(15%)	14	(13%)	0.055	10	(16%)	11	(17%)	0.044
Number of liver metastasis†	1	5	(4%)	6	(6%)		1	(2%)	5	(8%)	
	2	3	(2%)	3	(3%)		3	(5%)	2	(3%)	
	3	3	(2%)	4	(4%)		1	(2%)	3	(5%)	
	4	2	(2%)	0			1	(2%)	0		
	5	2	(2%)	0			2	(3%)	0		
	≥ 6	4	(3%)	1	(1%)		2	(3%)	1	(2%)	
cP	positive	42	(32%)	38	(36%)	0.075	21	(33%)	21	(33%)	0.000
Extent of peritoneal dissemination† (at initial diagnosis)	cP1a	14	(11%)	12	(11%)		7	(11%)	5	(8%)	
	cP1b	9	(7%)	3	(3%)		4	(6%)	2	(3%)	
	cP1c	15	(12%)	19	(18%)		9	(9%)	13	(20%)	
	cP1x	4	(3%)	4	(4%)		1	(2%)	1	(2%)	
cCY	positive	41	(32%)	29	(27%)	0.037	20	(31%)	22	(34%)	0.068
Clinical response											
	PD	3	(2%)	3	(3%)	0.042	1	(2%)	1	(2%)	0.000
	SD+ Non-CR/ non-PD	58	(45%)	53	(50%)	0.063	31	(48%)	32	(50%)	0.031
	CR + PR	69	(53%)	50	(47%)	0.075	32	(50%)	31	(48%)	0.031
	Missing	0		1	(1%)						
Operative year						0.491					0.066
	2011–2016	46	(35%)	61	(57%)		28	(44%)	30	(47%)	
	2017–2022	84	(65%)	46	(43%)		36	(56%)	34	(53%)	
Procedure						0.159					0.031
	TG	71	(55%)	66	(62%)		35	(55%)	34	(53%)	
	DG	53	(41%)	36	(34%)		26	(41%)	26	(41%)	
	PG	6	(5%)	4	(4%)		3	(5%)	3	(5%)	
	PD	0		1	(1%)		0	(0%)	1	(2%)	
Para-Aortic (distant) lymph node dissection		13	(10%)	35	(33%)	0.784	12	(19%)	11	(17%)	0.052
Combined resection (Major organ)		35	(27%)	58	(54%)	0.615	28	(44%)	29	(45%)	0.035
Combined resection (Esophagus)†		4	(3%)	6	(6%)		3	(5%)	4	(6%)	
Combined resection (DP)†		10	(8%)	11	(10%)		8	(13%)	5	(8%)	
Combined resection (Spleen)†		24	(18%)	40	(37%)		20	(31%)	18	(28%)	
Combined resection (Liver)†		5	(4%)	10	(9%)		2	(3%)	7	(11%)	
Combined resection (Colon)†		6	(5%)	10	(9%)		6	(9%)	4	(6%)	
Combined resection (Peritoneal dissemination)†		7	(17%)	14	(37%)		4	(6%)	3	(5%)	

Data are presented as median (interquartile range) for continuous variables, and numbers (%) for categorical variables

* For the Charlson Comorbidity Index, all patients had metastatic cancer; therefore, only comorbidities other than metastatic gastric cancer were counted

† The following variables were not included in the propensity score model: distant lymph node metastasis at initial diagnosis, number of liver metastases, extent of peritoneal dissemination at initial diagnosis, and combined resection of the esophagus, distal pancreas, spleen, liver, colon, and peritoneal dissemination

MIS, minimally invasive surgery; ASMD, absolute standardized mean difference; BMI, body mass index; ASA-PS, American Society of Anesthesiologists physical status; PD, progressive disease; SD, stable disease; CR, complete response; PR, partial response; TG, total gastrectomy; DG, distal gastrectomy; PG, proximal gastrectomy; PD, pancreaticoduodenectomy DP, distal pancreatectomy

### Preoperative chemotherapy

Table 2 summarises the preoperative chemotherapy in the matched cohort. The most common first-line regimens were doublet therapies with S-1/oxaliplatin (SOX) and S-1/cisplatin (SP), followed by triplet docetaxel/cisplatin/S-1 (DCS). The use of trastuzumab-containing regimens was approximately 10%, whereas that of immune checkpoint inhibitor (ICI)-containing regimens was < 5%. Total surgery duration and number of chemotherapy cycles did not differ significantly between the two groups. A summary of the preoperative chemotherapy regimens for the entire cohort is presented in Table S1.

**Table 2:** Summary of preoperative chemotherapy in the matched cohort

	MIS (*N* = 64)		Open (*N* = 64)	
First-line regimens				
Triplet				
DCS	14	(22%)	8	(13%)
DOS	2	(3%)	1	(2%)
DCF	1	(2%)	0	
SOX + PTX	0		1	(2%)
Doublet				
SOX	17	(27%)	24	(38%)
SP	19	(30%)	21	(33%)
CapeOX	2	(3%)	3	(5%)
Cape+ Cisplatin	4	(6%)	4	(6%)
FOLFOX	2	(3%)	1	(2%)
S-1	3	(5%)	1	(2%)
Trastuzumab-containing	9	(14%)	6	(9%)
Nivolumab-containing	2	(3%)	1	(2%)
Pembrolizumab-containing	1	(2%)	0	
IP-containing	0		2	(3%)
Total duration (days, IQR)	116	(97.5, 206.5)	129.5	(91, 206)
Total cycle				
≤ 3	37	(58%)	28	(44%)
4–10	18	(28%)	29	(45%)
≥ 11	9	(14%)	7	(11%)

MIS, minimally invasive surgery; DCS, docetaxel/ cisplatin/ S-1; DOS, docetaxel/ oxaliplatin/ S-1; DCF, docetaxel/ cisplatin/ 5-fluorouracil; SOX, S-1/ oxaliplatin; PTX, paclitaxel; SP, S-1/ cisplatin; CapeOX, capecitabine/ oxaliplatin; FOLFOX, 5-fluorouracil/ leucovorin/ oxaliplatin; IP, intraperitoneal chemotherapy; IQR, interquartile range

### Perioperative outcomes

The primary outcome, which was postoperative complications of CD grade ≥ II, occurred in 20(31%) and 30(47%) patients in the MIS and open surgery groups, respectively, with no significant difference observed (RR 0.67 [95% confidence interval [CI] 0.41–1.08]). Among postoperative complications, pancreatic fistula was slightly more common in the open surgery group, whereas anastomotic leakage was slightly more common in the MIS group. The most marked difference among the individual complications was seen in “other” complications, occurring in 1 case (2%) in the MIS group versus 9 cases (14%) in the open group. “Other” complications observed in the open surgery group included urinary tract infections, central venous catheter-related infections, and fevers of unknown origin. When limited to more severe complications (grade ≥ III), the incidence was comparable between the MIS (13%) and open surgery (16%) groups (RR 0.80, 95% CI 0.31–2.04), and there was no 30-day mortality. Operative time was significantly longer in the MIS group (MD 98 min, 95% CI 53–143), while blood loss volume was significantly lower (MD − 550 g, 95% CI − 738–−362). MIS achieved a higher proportion of R0 resections (92% vs. 78%, respectively, RR 1.18, 95% CI 1.02 − 1.37). R1 resections were more frequent in the open surgery group (6% vs. 17%, respectively). While all R1 cases in the MIS group were due to ypCY1, the open surgery group included several patients with positive gastric and distant metastatic resection margins (Table 3). Perioperative outcomes in the entire cohort (before PS matching) are presented in Table S2.

**Table 3:** Perioperative outcomes after operation in the matched population

	MIS (*N* = 64)		Open (*N* = 64)			Mean difference/ Risk ratio (95%CI)		
Complication grade ≥ 2	20	(31%)	30	(47%)		0.67	(0.41, 1.08)	
Incisional SSI	0		3	(5%)				
Leakage	5	(8%)	2	(3%)				
Pancreatic fistula	5	(8%)	8	(13%)				
Abscess	12	(19%)	15	(23%)				
Stricture	0		1	(2%)				
Bleeding	0		2	(3%)				
Ileus/ SBO	1	(2%)	2	(3%)				
DGE	1	(2%)	1	(2%)				
Pneumonia	2	(3%)	2	(3%)				
PE/DVT	2	(3%)	2	(3%)				
Ischemic heart disease	2	(3%)	0					
Others*	1	(2%)	9	(14%)				
Complication grade ≥ 3	8	(13%)	10	(16%)		0.8	(0.31, 2.04)	
30-day mortality	0		0			NE^‡^		
Operative time, minutes	422	(330–537)	329	(275–437)		98	(53, 143)	
Blood loss volume (g)	50	(5-165)	460	(259–1015)		-550	(-738, -362)	
Conversion to open surgery	2	(3%)	NA			NA		
Number of dissected lymph nodes	46	(33, 59)	37	(27, 49)		4.2	(−2.8, 11.3)	
Time to oral intake (days)	4	(4–6)	5	(4–6)		1.8	(−2.5, 6.1)	
Postoperative hospital stays (days)	14	(10–17)	17	(13–24)		−1.2	(−8.7, 6.4)	
30-day readmission after discharge	0	(0%)	2	(3%)		NE^‡^		
Reoperation	2	(3%)	1	(2%)		2.0	(0.18, 22.48)	
Residual tumor								
R0	59	(92%)	50	(78%)		1.18	(1.02, 1.37)	
R1†	4	(6%)	11	(17%)				
R2	1	(2%)	3	(5%)				

Continuous outcomes are shown as median (interquartile range) and mean difference, while categorical outcomes are shown as number (%) and risk ratio

*“Others” included one case of enteritis in the MIS group, and three cases of fever of unknown origin, two cases of urinary tract infection, one case of urinary retention, one case of central venous catheter infection, one case of diarrhea, and one case of cervical spondylosis in the open surgery group

†All R1 cases in the MIS group were due to ypCY1. In contrast, the open surgery group had 5 ypCY1 cases, 4 positive gastric resection margins, and 2 positive margins of resected distant metastases

‡Risk ratios were not estimable because no events occurred in one or both groups

MIS, minimally invasive surgery; SSI, surgical site infection; SBO, small bowel obstruction; DGE, delayed gastric emptying; PE, pulmonary embolism; DVT, deep venous thrombosis; NA, not applicable; NE, not estimable

### Pathological findings

At the time of pathological diagnosis, distant metastasis (ypM-positive) was more common in the open surgery group (MIS, 25% vs. open surgery, 50%, respectively). The proportion of postoperative chemotherapy administration was higher in the open surgery group (69% vs. 88%, respectively); however, the interval from surgery to postoperative chemotherapy was comparable between the two groups (median, 38 vs. 40 days, respectively). Even when limited to patients who achieved R0 resection, the proportion of patients who received adjuvant chemotherapy remained higher in the open surgery group (66% vs. 88%, respectively), whereas the interval to adjuvant chemotherapy was comparable between the groups (median, 38 vs. 39 days, respectively) (Table 4).

**Table 4:** Pathological findings and postoperative chemotherapy

		MIS (*N* = 64)		Open (*N* = 64)		Mean difference/ Risk ratio (95%CI)	
Lauren type							
	Intestinal	26	(41%)	19	(30%)		
	Diffuse	35	(55%)	39	(61%)		
	Missing	3	(5%)	6	(9%)		
ypT							
	≤ 3	42	(66%)	39	(61%)		
	4a	19	(30%)	19	(30%)		
	4b	3	(5%)	6	(9%)		
ypN							
	0	19	(30%)	19	(30%)		
	1–2	29	(45%)	20	(31%)		
	3	16	(25%)	25	(39%)		
ypM	positive	16	(25%)	32	(50%)		
ypH	positive	2	(3%)	6	(9%)		
ypP	positive	5	(8%)	13	(20%)		
ypCY	positive	4	(6%)	6	(9%)		
Paraaortic lymph node							
	localized in a2/b1	3	(5%)	6	(9%)		
	within a1/b2	0	(0%)	3	(5%)		
	far from a1/b2	1	(2%)	0	(0%)		
Final stage							
	0	3	(5%)	2	(3%)		
	I	10	(16%)	3	(5%)		
	II	12	(19%)	15	(23%)		
	III	23	(36%)	12	(19%)		
	IV	16	(25%)	32	(50%)		
Pathological response (Grade*)							
	0	2	(3%)	4	(6%)		
	1	29	(45%)	32	(50%)		
	2	18	(28%)	17	(27%)		
	3	4	(6%)	3	(5%)		
	Missing	11	(17%)	8	(13%)		
Postoperative chemotherapy		44	(69%)	56	(88%)	0.79	(0.65, 0.95)
Interval from operation to postoperative chemotherapy (days)		38	(32, 46)	40	(27, 55)	−7.2	(−16.4, 2.0)
Adjuvant chemotherapy (Among R0 achievers)		39/ 59	(66%)	44/ 50	(88%)	0.75	(0.61, 0.93)
Interval from operation to Adjuvant chemotherapy (days) (Among R0 achievers)		38	(32, 46)	39	(26.5, 50)	−7.7	(−18.7, 3.3)

Continuous variables are shown as median (interquartile range), and categorical variables are shown as number (%)

We additionally reported the risk ratio for the administration of postoperative chemotherapy and the mean difference for the day from operation to chemotherapy

The final stage and pathological response were determined according to the Japanese Classification of Gastric Carcinoma, 15th edition

*Grade 0, 100% residual tumor, Grade 1, > 1/3; Grade 2, < 1/3; Grade 3, 0%

MIS, minimally invasive surgery

### Long-term outcomes

**Fig. 2:**
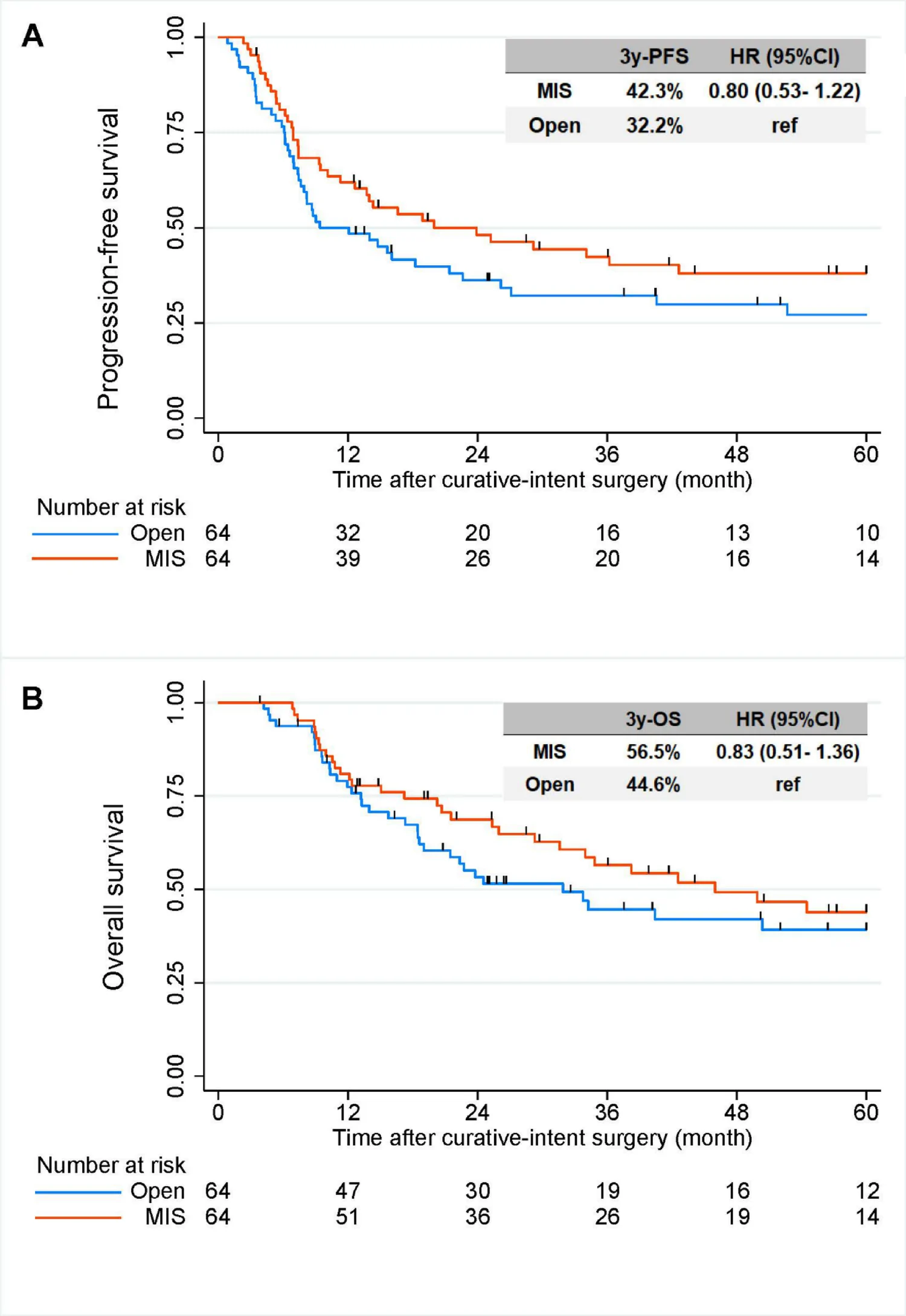
Kaplan–Meier survival curves in the matched cohort **A** progression-free survival, **B** overall survival. HR, hazard ratio; MIS, minimally invasive surgery; 3y-OS, 3 year-overall survival; 3y-PFS, 3 year-progression-free survival; ref, reference

The Kaplan–Meier curves for PFS and OS in the matched cohort are shown in Fig. 2. The median follow-up periods among survivors for the matched population were 41.4 months in the MIS group and 40.2 months in the open surgery groups. The 3-year PFS was 42.3% in the MIS group and 32.2% in the open surgery group (HR 0.80, 95% CI: 0.53–1.22) (Fig. 2A). The 3-year OS was 56.5% in the MIS group and 44.6% in the open surgery group, while the median survival time (MST) was 46.0 months vs. 31.9 months, respectively (HR 0.83, 95% CI 0.51–1.36) (Fig. 2B). As there was an imbalance in the distribution of distant metastases and R0 status confirmed by pathological or final diagnosis between the two groups, additional analyses of PFS and OS stratified according to ypM (positive/negative) or R0 status (R0/non-R0) were performed. Among the patients who were ypM-positive, those in the MIS group showed a slightly more favorable outcome (HR for PFS: 0.51, 95% CI 0.26–1.01; HR for OS: 0.53, 95% CI 0.23–1.19) (Figure S2A, B), whereas among the patients who were ypM-negative, the Kaplan-Meier curves were nearly overlapping (HR for PFS: 1.35, 95% CI 0.73–2.50; HR for OS: 1.40, 95% CI 0.70–2.81) (Figure S2C, D). However, even among the patients who were ypM-positive, a difference remained in the R0 status between the groups. When limited to the patients who achieved R0 resection, both groups showed comparable outcomes (HR for PFS: 1.02, 95% CI 0.62–1.68; HR for OS: 1.04, 95% CI 0.57–1.92) (Figure S3A, B). Regarding non-R0 cases, although the number of patients was small, PFS was significantly prolonged in the MIS group, with OS also showing a favorable trend in this group (Figure S3C, D). The peritoneum was the most common site of recurrence or progression, followed by the lymph nodes and liver. No significant difference was observed in the patterns of initial recurrence or progression between the two groups (Table S3).

### Subgroup analysis

In a subgroup analysis of older individuals (aged ≥ 75 years), those with macroscopic non-type 4, good clinical response (CR/PR), cT ≤ 3, cCY0, and those undergoing non-TG and no combined dissection, MIS was significantly associated with a reduced incidence of CD grade ≥ II complications (Fig. 3A). No difference in trends regarding complications was observed between the ypM-positive and ypM-negative subgroups. Across all subgroup analyses of OS, no significant differences were observed between the MIS and open surgery groups (Fig. 3B).

Fig. 3Subgroup analysis for **A** complication grade ≥ II and **B** overall survival. MIS, minimally invasive surgery; SD, Stable Disease; CR, complete response; PD, progressive disease; PR, partial response; TG, total gastrectomy; PAND, para-aortic lymph node dissection; NAC, neoadjuvant chemotherapy; *NE*,* not estimable*

**Figure:**
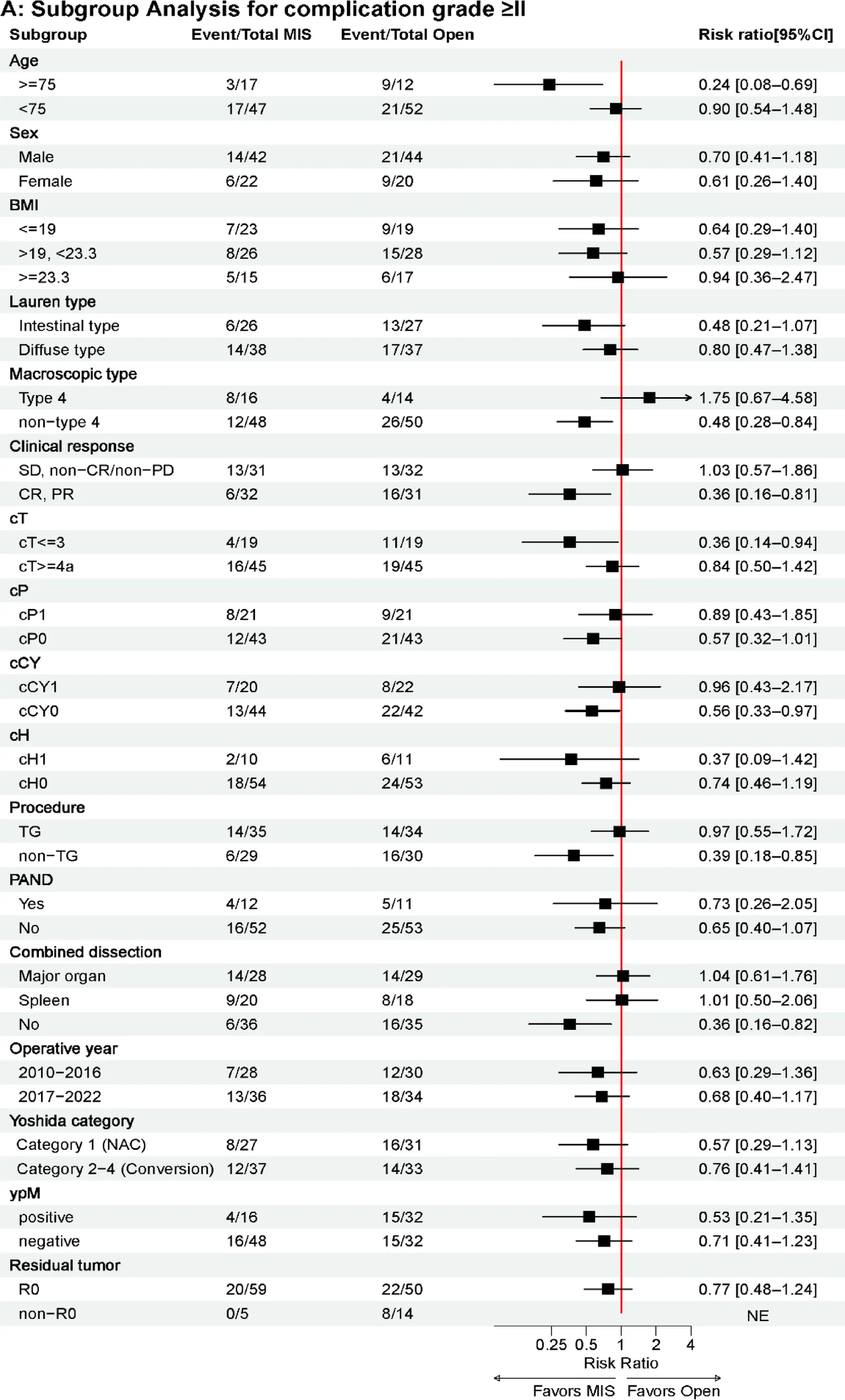

**Figure:**
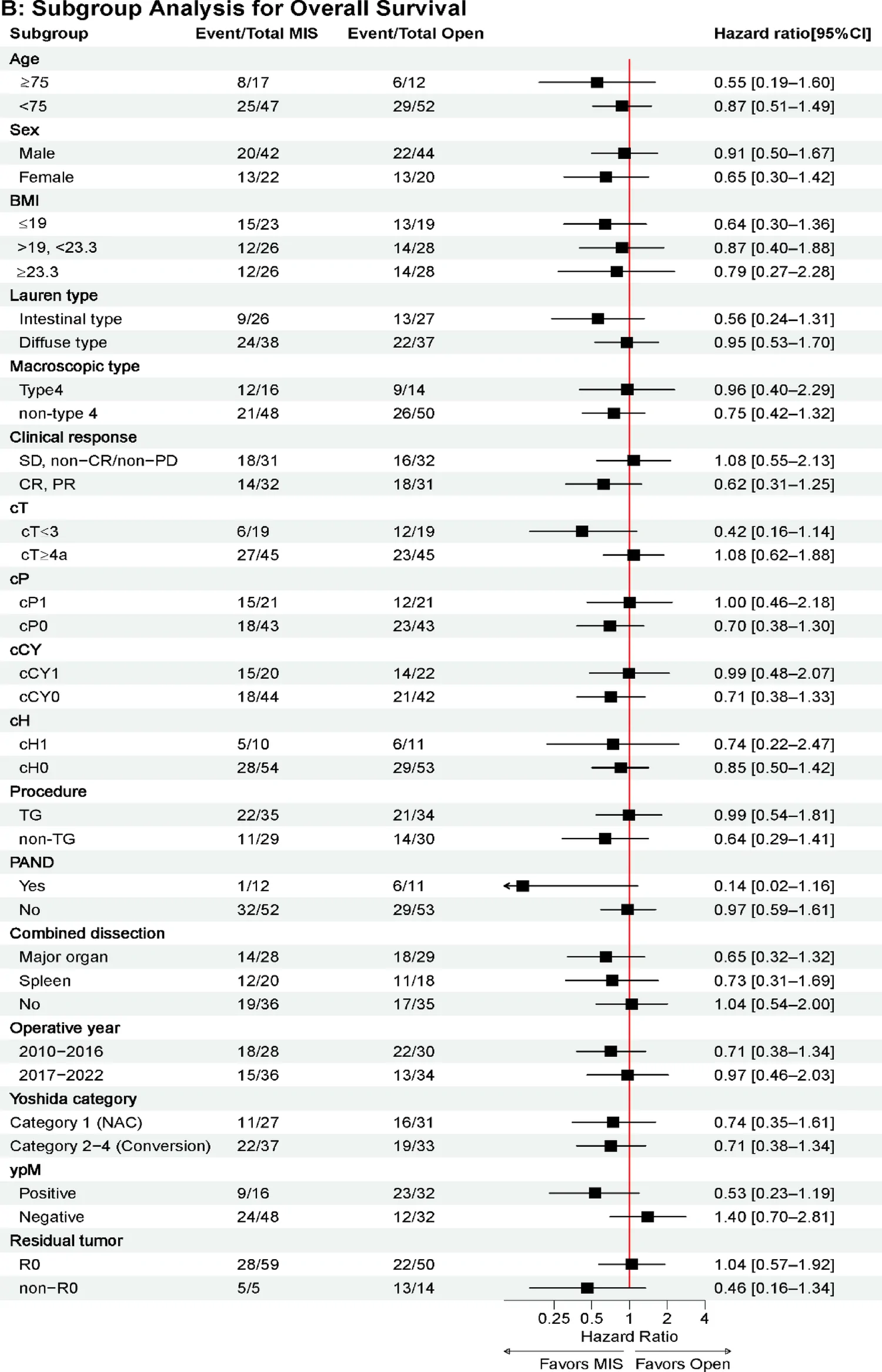


### Sensitivity analysis

In sensitivity analyses of postoperative grade ≥ II complications, trends were consistent with the main analysis. MIS was significantly associated with reduced complications in the PS matching including preoperative chemotherapy duration, the IPTW with average treatment effect on the treated (ATT) weighting, and all analyses included institutional factors. Sensitivity analyses for OS showed trends similar to those in the main analysis (Figure S4).

## Discussion

Our findings indicated that no significant difference in perioperative safety or long-term survival were observed between MIS and open surgery in curative-intent surgery after chemotherapy for stage IV GC. Consistent with previous studies, MIS had a significantly longer operative time, but markedly reduced blood loss. Considering the findings of the primary analysis and the statistically significant reduction in complications observed in several sensitivity analyses, MIS may be regarded as a safe approach. Given the limited number of patients with stage IV GC who undergo curative-intent surgery after chemotherapy, prospective randomised trials are unlikely to be feasible in this setting. In this multicenter study, we assembled one of the largest reported cohorts, maintained surgeon quality through a collaborative study group, and carefully adjusted for potential confounders, including the operative era, thereby enhancing the reliability of our results. It should be noted that our cohort included not only patients in the conversion setting—those initially diagnosed unresectable who underwent curative-intent surgery after a favorable response to systemic chemotherapy—but also patients in the neoadjuvant chemotherapy (NAC) setting, who were considered potentially resectable at diagnosis and received preoperative chemotherapy for oligometastasis prior to curative resection. Our findings provide high-quality evidence to guide the surgical management of stage IV GC in the era of modern multimodal therapy.

We observed a trend towards a lower incidence of CD grade ≥ II complications in the MIS group, although not statistically significant. This trend may reflect the benefits of earlier postoperative mobilization resulting from reduced invasiveness of MIS, because the higher incidence of complications in the open surgery group was related to prolonged intravenous or urinary catheter use. Given the imbalance in ypM status between the two groups, subgroup analyses were conducted; however, the results were consistent with the primary analysis, suggesting minimal influence. In contrast, among the patients with macroscopic type 4 tumors, the open surgery group showed relatively favorable outcomes. While this finding was somewhat inconsistent with previous reports, it possibly reflects the technical difficulty in manipulating large, diffuse tumors with laparoscopic instruments [38–40]. Conversely, in patients with a favorable chemotherapy response (CR/PR), MIS may offer advantages such as enhanced magnification and delicate dissection techniques, which may offset chemotherapy-induced fibrosis and loss of dissection planes, thereby contributing to fewer complications. These technical advantages in MIS may also have played a role in reducing R1 resection rates through fewer positive resection margins. With the increasing adoption of ICIs and their favorable response rates, future investigations should clarify their potential cooperation with MIS.

In terms of long-term outcomes, MIS showed no significant differences compared with open surgery. Similar trends were seen in most subgroup and sensitivity analyses, supporting the validity of our results. The open surgery group included more patients who were ypM-positive, which may suggest an imbalance in the baseline prognosis. However, subgroup analyses restricted to ypM-negative or R0-resected populations with higher comparability between the two approaches confirmed equivalent long-term outcomes. Regarding preoperative chemotherapy, the proportion of patients receiving triplet regimens was higher in the MIS group, particularly in the DCS regimen; however, previous studies have reported no significant differences in outcomes between SP and DCS regimens [6, 41]. Furthermore, analysis including the duration of preoperative chemotherapy showed results generally consistent with the main analysis, suggesting that the influence of preoperative chemotherapy on survival outcomes is likely minimal. Postoperative chemotherapy should also be considered when interpreting the survival results. In this study, postoperative chemotherapy was more frequently administered in the open surgery group even when limited to R0 cases. Current guidelines do not provide clear recommendations for postoperative chemotherapy following conversion surgery, and in our cohort, the variation in administration largely reflected the physician’s discretion [42]. This may have introduced heterogeneity in treatment intensity after surgery. Because patients with clinically unfavorable features may have been more likely to receive intensive chemotherapy, postoperative treatment could have modified subsequent survival outcomes. Thus, we cannot exclude the possibility that differences in postoperative chemotherapy contributed to the lack of a significant survival difference between the two surgical groups.

In this study, the 3-year OS rates were 56.5% and 44.6% for MIS and open surgery, respectively, with MSTs of 46.0 and 31.9 months, respectively. These outcomes are comparable to the 3-year OS rate of 59% reported in the JCOG0405 and MST of 36.7 months in the CONVO-GC-1 cohort, which provides supporting external validity for our findings [7, 43]. While advances in targeted agents and ICIs have extended survival in metastatic disease, the median OS generally remains approximately 20 months [44–48]. Our results reaffirm that curative surgery after chemotherapy can provide meaningful survival benefits for a subset of patients with stage IV GC. Despite the role of conversion surgery remaining controversial, there is little doubt that R0 resection can offer durable survival in carefully selected patients [4–8]. Furthermore, the incorporation of robotic surgery and ICI-based regimens for part of our cohort reflects evolving clinical practice, underscoring the need for ongoing accumulation of cases to clarify the role of MIS within multimodal treatment strategies.

This study had some limitations. The open surgery group may have included patients with inherently poor prognoses. Only curative-intent surgeries were analysed; however, our study’s retrospective design made it difficult to fully verify surgical intent. Some surgeons, as well as institutional policies, may favor more aggressive resection of metastatic lesions in an open setting. Moreover, some surgeons may have attempted open surgery without prior staging laparoscopy and proceeded with the operation despite intraoperative detection of positive cytology or metastasis. In gastric cancer, where discrepancies between clinical staging and pathological staging are often observed, these reasons may have contributed to the imbalance in ypM status and R1 resection between the two groups [49]. In addition, the administration of ICIs and molecular targeted agents during preoperative chemotherapy differed slightly between the groups, and postoperative chemotherapy regimens and durations were not uniformly captured, potentially introducing bias. These limitations are inherent to retrospective studies; thus, a randomised controlled trial would be ideal for this population although it would be logistically challenging. Furthermore, some subgroup analyses (especially H1, para-aortic lymph node dissection, and non-R0 cases) had small sample sizes, leading to limited statistical power and requiring cautious interpretation of the results. Finally, because chemotherapy backbones differ between regions (e.g. limited use of 5-fluorouracil, leucovorin, oxaliplatin, and docetaxel [FLOT] in Japan) and obesity was less prevalent in our cohort, generalisability to Western populations should be considered with caution [50].

In conclusion, MIS did not demonstrate significant differences in either short- or long-term outcomes compared with open surgery; however, the results were not poor. Although the impact of surgical approach on outcomes is likely smaller than that of factors such as tumor biology and response to drug therapy, these findings suggest that, given its minimally invasive nature, MIS may be a feasible treatment option for stage IV GC after chemotherapy when performed with curative-intent by experienced surgeons.

## Supplementary information

Below is the link to the electronic supplementary material.


Supplementary material 1


## Data Availability

The datasets generated and analysed during the current study are not publicly available but are available from the corresponding author upon reasonable request.
