# Supplementary material for: Comparison of minimally invasive surgery and open surgery following chemotherapy for cStage IVb gastric cancer: a multicenter retrospective cohort study
Source: Gastric Cancer. 2026 Jul 20;29(5):1150–65. doi: 10.1007/s10120-026-01779-y (PMC13525005; doi:10.1007/s10120-026-01779-y)
Supplement: Supplementary file 1 [file 10120_2026_1779_MOESM1_ESM.docx]

**Comparison of minimally invasive surgery and open surgery following chemotherapy for cStage IVb gastric cancer: a multicenter retrospective cohort study**

Yusuke Fujii, MD^1,2^, Shigeo Hisamori, MD, PhD, FACS^1,2,*^, Nobuaki Hoshino, MD, PhD, MPH^1,2^, Seiichiro Kanaya, MD, PhD, FACS^2,3^, Eiji Tanaka, MD, PhD^2,4^, Yoshito Yamashita, MD, PhD^2,5^, Akira Miki, MD^2,6^, Kosuke Toda, MD, PhD^2,7^, Michihiro Yamamoto, MD^2,8^ Yosuke Kinjo, MD, PhD^2,9^, Dai Manaka, MD, PhD^2,10^, Hiroaki Hata, MD, PhD^2,11^, Hironori Kawada, MD, PhD^2,12^, Shotaro Matsuda, MD^2,13^, Atsushi Itami, MD, PhD, FACS^2,14^, Kyoichi Hashimoto, MD, PhD^2,15^, Kenjiro Hirai, MD, PhD^2,16^, Sanae Nakajima, MD, PhD^2,17^, Hiroshi Okabe, MD, PhD, FACS^2,18^, Masazumi Sakaguchi, MD, PhD^1,2,19^, Yuichiro Kawamura, MD, PhD^2,20^, Takashi Sakamoto, MD, PhD^1,2^, Shintaro Okumura, MD, PhD^1,2^, Tatsuto Nishigori, MD, PhD, FACS^1,2,19^, Shigeru Tsunoda, MD, PhD, FACS^1,2^, and Kazutaka Obama, MD, PhD, FACS^1,2^

1. Department of Surgery, Kyoto University Hospital, Kyoto, Japan

2. Kyoto Esophageal and Gastric Surgery Group, Japan

3. Department of Gastroenterological Surgery, Japanese Red Cross Osaka Hospital, Osaka, Japan

4. Department of Gastroenterological Surgery, Kitano Hospital, Osaka, Japan

5. Department of Gastroenterological Surgery, Japanese Red Cross Wakayama Medical Center, Wakayama, Japan

6. Department of Gastroenterological Surgery, Toyooka Hospital, Toyooka, Japan

7. Department of Surgery, Shiga General Hospital, Moriyama, Japan

8. Department of Gastroenterological Surgery, Tenri Hospital, Tenri, Japan

9. Department of Gastroenterological Surgery and Oncology, Himeji Medical Center, Himeji, Japan

10. Department of Surgery, Gastrointestinal Center, Kyoto Katsura Hospital, Kyoto, Japan

11. Department of Surgery, National Hospital Organization, Kyoto Medical Center, Kyoto, Japan

12. Department of Gastroenterological Surgery, Hyogo Prefectural Amagasaki General Medical Center, Amagasaki, Japan

13. Department of Surgery and Transplant Surgery, Kobe City Medical Center General Hospital, Kobe, Japan

14. Department of Surgery, Kobe City Nishi-Kobe Medical Center, Kobe, Japan

15. Department of Gastroenterological Surgery, Uji-Tokushukai Medical Center, Uji, Japan

16. Department of Surgery, Japanese Red Cross Otsu Hospital, Otsu, Japan

17. Department of Gastroenterological Surgery, Kobe City Medical Center West Hospital, Kobe, Japan

18. Department of Gastroenterological Surgery, New Tokyo Hospital, Matsudo, Japan

19. Department of general Surgery, Kyoto City Hospital, Kyoto, Japan

20. Department of Surgery, Kokura Memorial Hospital, Kitakyushu, Japan

***Corresponding author:** Shigeo Hisamori

Address: 54 Shogoin Kawahara-cho, Sakyo-ku, Kyoto, 606-8507, Japan

Phone number: [+81-(0)75-366-7595](tel:%2B81-%280%2975-366-7595)

FAX number: +81-(0)75-366-7642

Email address: [hisamori@kuhp.kyoto-u.ac.jp](mailto:hisamori@kuhp.kyoto-u.ac.jp)

**Supplementary Materials - Index**

| **Supplementary Tables** |  | |
| --- | --- | --- |
| **Table S1:** Summary of preoperative chemotherapy in the entire cohort (before PS matching) | *pag. 4* | |
| **Table S2:** Perioperative outcomes after operation in the entire cohort (before PS matching) | *pag. 5-6* | |
| **Table S3:** Types of the first recurrence/ progression | *pag. 7* | |
| **Supplementary Figures** |  | |
| **Figure S1:** The trend of approach selection during the study period | *pag. 8* | |
| **Figure S2:** Kaplan-Meier survival curves stratified by ypM status | | *pag. 9-10* |
| **Figure S3:** Kaplan-Meier survival curves stratified by R0 status | | *pag. 11-12* |
| **Figure S4:** Sensitivity analysis | | *pag. 13* |
|  | |  |
|  | |  |
|  |  | |
|  |  | |

**Supplementary Tables**

**Table S1:** Summary of preoperative chemotherapy in the entire cohort (before PS matching)

|  | MIS (N=130) | | Open (N=107) | |
| --- | --- | --- | --- | --- |
| **First-line regimens** |  |  |  |  |
| Triplet |  |  |  |  |
| DCS | 21 | (16%) | 14 | (13%) |
| DOS | 2 | (2%) | 2 | (2%) |
| DCF | 1 | (1%) | 0 |  |
| SP+PTX | 0 |  | 1 | (1%) |
| SOX+PTX | 0 |  | 1 | (1%) |
| Doublet |  |  |  |  |
| SOX | 51 | (39%) | 32 | (30%) |
| SP | 37 | (28%) | 44 | (41%) |
| CapeOX | 3 | (2%) | 3 | (3%) |
| Cape+Cisplatin | 7 | (5%) | 7 | (7%) |
| FOLFOX | 3 | (2%) | 1 | (1%) |
| DTX+S-1 | 1 | (1%) | 0 |  |
| 5-FU+Cisplatin | 1 | (1%) | 1 | (1%) |
|  |  |  |  |  |
| S-1 | 3 | (2%) | 1 | (1%) |
|  |  |  |  |  |
| Trastuzumab-containing | 17 | (13%) | 11 | (10%) |
| Nivolumab-containing | 5 | (4%) | 1 | (1%) |
| Pembrolizumab-containing | 1 | (1%) | 0 |  |
| IP-containing | 0 |  | 2 | (2%) |
|  |  |  |  |  |
| **Total duration, day (IQR)** | 119 | (101, 191) | 103 | (85, 172) |
| **Total cycle** |  |  |  |  |
| ≤3 | 66 | (51%) | 60 | (56%) |
| 4-10 | 50 | (39%) | 36 | (34%) |
| ≥11 | 14 | (11%) | 11 | (10%) |

PS, propensity score; MIS, minimally invasive surgery; DCS, docetaxel/ cisplatin/ S-1; DOS, docetaxel/ oxaliplatin/ S-1; DCF, docetaxel/ cisplatin/ 5-fluorouracil; SP, S-1/ cisplatin; PTX, paclitaxel; SOX, S-1/ oxaliplatin; CapeOX, capecitabine/ oxaliplatin; FOLFOX, 5-fluorouracil/ leucovorin/ oxaliplatin; DTX, docetaxel; 5-FU, 5-fluorouracil; IP, intraperitoneal chemotherapy; IQR, interquartile range

Table S2. Perioperative outcomes after operation in the entire cohort (before PS matching)

|  | MIS (N=130) | | Open (N=107) | | Mean difference/ Risk ratio (95%CI) | | |  |
| --- | --- | --- | --- | --- | --- | --- | --- | --- |
| Complication grade ≥2 | 42 | (32%) | 55 | (51%) | | 0.63 | (0.46, 0.86) | |
| Incisional SSI | 2 | (2%) | 4 | (4%) | |  |  | |
| Leakage | 7 | (5%) | 3 | (3%) | |  |  | |
| Pancreatic fistula | 6 | (5%) | 13 | (12%) | |  |  | |
| Abscess | 20 | (15%) | 23 | (22%) | |  |  | |
| Stricture | 0 |  | 1 | (1%) | |  |  | |
| Bleeding | 1 | (1%) | 4 | (4%) | |  |  | |
| Ileus/ SBO | 2 | (2%) | 4 | (4%) | |  |  | |
| DGE | 5 | (4%) | 2 | (2%) | |  |  | |
| Pneumonia | 7 | (5%) | 6 | (6%) | |  |  | |
| PE/DVT | 3 | (2%) | 4 | (4%) | |  |  | |
| Ischemic heart disease | 2 | (2%) | 0 |  | |  |  | |
| Others | 6 | (5%) | 19 | (18%) | |  |  | |
| Complication grade ≥3 | 12 | (9%) | 15 | (14%) | | 0.66 | (0.32, 1.35) | |
| 30-day mortality | 0 |  | 0 |  | | NE* |  | |
| Operative time, minutes | 417 | (325-534) | 336 | (277-455) | | 77 | (41, 112) | |
| Blood loss volume (g) | 54 | (5-150) | 590 | (296-1104) | | -632 | (-763, -500) | |
| Conversion to open surgery | 2 | (2%) | NA |  | | NA |  | |
| Number of dissected lymph nodes | 42 | (31, 56) | 39 | (29, 51) | | 0.3 | (-4.8, 5.3) | |
| Time to oral intake (days) | 4 | (3-5) | 5 | (4-6) | | 0 | (-2.4, 2.4) | |
| Postoperative hospital stays (days) | 14 | (10-17) | 17 | (13-24) | | -3.0 | (-7.7, 1.7) | |
| 30-day readmission after discharge | 2 | (2%) | 6 | (6%) | | 0.27 | (0.06, 1.33) | |
| Reoperation | 6 | (5%) | 4 | (4%) | | 1.23 | (0.36, 4.26) | |
| Residual tumor |  |  |  |  | |  |  | |
| R0 | 121 | (93%) | 82 | (77%) | | 1.21 | (1.08, 1.36) | |
| R1 | 6 | (5%) | 19 | (18%) | |  |  | |
| R2 | 3 | (2%) | 6 | (6%) | |  |  | |

Continuous outcomes are shown as median (interquartile range) and mean difference, while categorical outcomes are shown as number (%) and risk ratio.

*Risk ratios were not estimable because no events occurred in both groups.

PS, propensity score; MIS, minimally invasive surgery; SSI, surgical site infection; SBO, small bowel obstruction; DGE, delayed gastric emptying; PE, pulmonary embolism; DVT, deep venous thrombosis; NA, not applicable; NE, not estimable

**Table S3:** Types of the first recurrence/ progression

| Recurrence/ Progression | MIS (N=64) | | Open (N=64) | |
| --- | --- | --- | --- | --- |
| Peritoneal | 25 | (39%) | 21 | (33%) |
| Hematogenous | 10 | (16%) | 14 | (22%) |
| Liver | 7 | (11%) | 8 | (13%) |
| Lung | 2 | (3%) | 0 | (0%) |
| Lymph node | 10 | (16%) | 12 | (19%) |
| Anastomotic site | 1 | (2%) | 3 | (5%) |
| Other* | 0 | (0%) | 1 | (2%) |
| Total | 41 | (64%) | 44 | (69%) |

Data are presented as numbers (%).

The list contains duplicates.

*“Other” included one case of malignant pleural effusion in the open surgery group.

MIS, minimally invasive surgery

**Supplementary Figures**

**Figure S1:** The trend of approach selection during the study period

|  | Open | Laparoscopic | Robotic |
| --- | --- | --- | --- |
| 2011 | 11 | 4 | 0 |
| 2012 | 10 | 6 | 1 |
| 2013 | 14 | 6 | 1 |
| 2014 | 9 | 15 | 0 |
| 2015 | 18 | 4 | 1 |
| 2016 | 8 | 9 | 0 |
| 2017 | 8 | 13 | 0 |
| 2018 | 11 | 16 | 4 |
| 2019 | 4 | 6 | 2 |
| 2020 | 10 | 7 | 8 |
| 2021 | 9 | 5 | 7 |
| 2022 | 5 | 9 | 8 |

**Figure S2:** Kaplan-Meier survival curves stratified by ypM status

(A) Progression-free survival and (B) Overall survival among ypM-positive patients.

(C) Progression-free survival and (D) Overall survival among ypM-negative patients.

3y-PFS, 3 year-progression-free survival; HR, hazard ratio; MIS, minimally invasive surgery; ref, reference; 3y-OS, 3 year-overall survival

**Figure S3:** Kaplan-Meier survival curves stratified by R0 status

(A) Progression-free survival and (B) Overall survival among R0 patients.

(C) Progression-free survival and (D) Overall survival among non-R0 patients.

3y-PFS, 3 year-progression-free survival; HR, hazard ratio; MIS, minimally invasive surgery; ref, reference; 3y-OS, 3 year-overall survival

**Figure S4: Sensitivity analysis**

Sensitivity analysis for (A) complication grade≥ II and (B) overall survival.

The first rows present the results of the main analysis for comparison.

PSM, propensity score matching; PD, pancreaticoduodenectomy; IPTW, inverse probability of treatment weighting; ATT, average treatment effect on the treated; GEE, generalized estimating equations; MI, multiple imputation; MIS, minimally invasive surgery
